# Validation of the Chinese version of the Brief Pain Inventory in patients with knee osteoarthritis

**DOI:** 10.1186/s13018-023-04218-1

**Published:** 2023-09-23

**Authors:** Shunxing Wang, Shuxin Yao, Lei Shang, Chao Xu, Jianbing Ma

**Affiliations:** 1 Department of Knee Joint Surgery, Honghui Hospital, Xi’an Jiaotong University, No. 555 E.Youyi Rd, Xi’an, 710061 China; 2 Xi’an medical university, No. 1, Xinwang Rd, Weiyang District, Xi ‘an, 710021 China; 3 Department of Health Statistics, Faculty of Preventive Medicine, The Air Force Military Medical University, No.169 W. Changle Rd, Xi’an, 710032 China

**Keywords:** Brief Pain Inventory, Knee osteoarthritis, Pain measurement, Chinese, Psychometric properties

## Abstract

**Background:**

Knee osteoarthritis (KOA) primarily presents with symptoms of pain and compromised functionality. Pain is a subjective manifestation that necessitates the employment of reliable evaluation tools for practical assessment, thereby enabling the formulation of appropriate interventional strategies. The Brief Pain Inventory (BPI) is a widely utilized questionnaire for evaluating the status of chronic pain. The purpose of the present study is to translate the short form of BPI into Chinese version (BPI-CV) and conduct cross-cultural adaptation to evaluate the psychometric characteristics of BPI-CV in KOA patients.

**Methods:**

BPI-CV was translated and cross-culturally adapted according to internationally recognized guidelines. A cohort comprising 150 patients diagnosed with KOA successfully completed the demographic questionnaire, BPI-CV, Western Ontario and McMaster University Osteoarthritis Index (WOMAC), and the EuroQoL Group's five-dimension questionnaire (EQ-5D). Internal consistency and test–retest analysis were used to evaluate the reliability. The internal consistency of the scale items was evaluated by calculating the Cronbach's *α* value (> 0.7). We chose to employ two scales commonly used in the evaluation of KOA patients: the disease-specific WOMAC scale and the universal EQ-5D scale. Construct validity was determined through Pearson correlation analysis, comparing BPI scores with those obtained from the WOMAC and EQ-5D scales. Exploratory factor analysis was used to structural validity.

**Results:**

The BPI-CV was well accepted with no ceiling or floor effect. Cronbach's *α* for assessing internal consistency was 0.894. Test–retest reliability was excellent with an ICC of 0.852 (95%CI 0.785–0.905). The BPI-CV showed moderate to strong correlations with the pain dimension (*r* = 0.496–0.860) and the functional interference dimension (*r* = 0.517–0.712) of the WOMAC and the EQ-5D (*r* = 0.527–0.743). Three factors resulted using exploratory factor analysis: pain severity, activity interference, and emotional interference, accounting for 79.0% of the total variance. Standard error of measurement was 0.539.

**Conclusion:**

BPI-CV has good feasibility, reliability, and validity. It can be recommended for KOA patients in mainland China.

**Supplementary Information:**

The online version contains supplementary material available at 10.1186/s13018-023-04218-1.

## Introduction

Knee osteoarthritis (KOA) is one of the most common chronic diseases [1, 2]. Pain is the main factor that drives patients to pay attention to medical treatment, and it is also the main factor that leads to functional limitation and quality of life decline [3]. In addition, pain may be the most important variable in deciding whether to operate [4]. Therefore, the measurement of pain is important in clinical practice [5]. However, since pain is a subjective symptom and cannot be reliably measured through external evaluation, effective and reliable measurement tools are needed to evaluate the patients’ subjective perception on pain [6]. Patient-reported outcome measures scale (PROMs) is now widely accepted as the gold standard for pain evaluation [7].

The Brief Pain Inventory (BPI) is an validated, reliable, and commonly used instrument that can assess the location and severity of pain and its impact on pain individuals [6]. BPI was originally invented for cancer patients and has been adapted into multiple languages [8–12]. There is already a Chinese version of BPI for cancer patients [13]. However, its psychological characteristics for nonmalignant pain have not been verified in China, especially for KOA patients.

Currently, English and Norwegian studies have reported that BPI has favorable psychometric properties in osteoarthritis patients [4, 14]. As far as we know, there is no study showing the psychometric characteristics of BPI in Chinese KOA patients. Therefore, the purpose of the present study was to translate and adapt the short form of BPI cross-culturally into a simplified Chinese version (BPI-CV) and verify its reliability and validity in KOA patients.

## Methods

### Translation and cross-cultural adaptation

The five steps of translation and cross-cultural adaptation follow the previous guidelines [15]. In the first step of this process, two bilingual translators, one being an orthopedic doctor and the other being a professional translator without a medical background, independently translated the BPI from English to simplified Chinese. Subsequently, in the second step, a consensus meeting involving the two translators and several authors of this paper was convened to reconcile discrepancies arising from linguistic expressions and cultural nuances, leading to the creation of the initial BPI-CV. In the third step, two bilingual translators with medical expertise independently translated this initial BPI-CV back into English, reaching a consensus in the process. Moving on to the fourth step, a consensus meeting was conducted with all researchers involved to address any disparities, ambiguities, or oral concerns, ultimately culminating in the final BPI-CV. Finally, in the fifth step, 50 KOA diagnosed patients were invited by researchers to conduct preliminary testing of the final version, collecting valuable feedback for further refinements. Following thorough discussions among all researchers to address issues encountered during pre-testing, the ultimate and definitive BPI-CV was developed (as detailed in Additional file 1: Appendix A).

### Patients and data collection

According to the rule of 10 patients for each scale item, at least 110 participants are required to fully evaluate the reliability parameters [16]. From January 2022 to January 2023, 150 patients diagnosed as KOA were recruited from the outpatient of joint surgery, Honghui Hospital in Shaanxi Province.

The inclusion criteria of the present study are as follows: (a) age > 18 years old; (b) the cognitive level can meet the requirements of filling in the questionnaire; and (c) fluent Mandarin at conversational level. Exclusion criteria are as follows: (a) history of other vascular, neural, and musculoskeletal diseases that affect activities or produce pain symptoms and (b) serious diseases that affect daily life, such as heart disease, dyspnea, psychosis, etc. All involved participants signed the informed consent form, and the medical ethics committee of Honghui Hospital approved this prospective observational study (No. 202212003).

### Instruments

#### Demographic information

Each participant must complete a general demographic information questionnaire, including age, gender, height and weight (calculated body mass index (BMI)), education level, employment status, living conditions, and pain duration, and then complete three questionnaires, including BPI-CV, WOMAC, and EQ-5D. All questionnaires are managed by well-trained interviewers.

### The short form of Brief Pain Inventory (BPI)

BPI is a short, self-management questionnaire. BPI has two main scores: pain severity score and pain interference score. The pain severity score is calculated according to four items of pain intensity, which are, respectively, the "most severe," "at least," and "average" pain of the patient in the past 24 h and the "current pain" (the pain when completing the questionnaire). The score of each item ranges from 0 = "no pain" to 10 = "the most severe pain you can imagine" and contributes to the final score with the same weight, ranging from 0 to 40. The pain interference score is calculated according to the seven subitems of the pain interference item, which include assessing the interference degree of their pain on "life enjoyment," "general activities," "walking ability," "emotion," "sleep," "normal work," and "relationship with other people." The seven subitems are rated from 0 = "no interference" to 10 = "complete interference," ranging from 0 to 70 [17].

### The Western Ontario and McMaster Universities Osteoarthritis Index (WOMAC)

The WOMAC stands as a validated and reliable measure extensively employed for evaluating individuals afflicted by osteoarthritis [18]. Its widespread adoption spans clinical trials, investigative inquiries, and medical applications, serving as a pivotal tool in monitoring the advancement of the condition, gauging the efficacy of interventions, and steering the course of therapeutic choices. The scale consists of three dimensions: stiffness (2 items), pain (5 items), and joint function (17 items). Each item is recoded into a 0–10 scale, in which 10 represents the largest problem, and 0 represents no problem [19].

### The EuroQoL Group's five-dimension questionnaire (EQ-5D)

EQ-5D is a multi-dimensional health-related quality of life (HRQoL) measurement scale [20]. The EQ-5D instrument has demonstrated commendable reliability and validity, establishing it as a widely accepted tool for gauging HRQOL within the Chinese population [21]. The scale describes the health status from five dimensions: mobility, self-care, usual activities, pain/discomfort, and anxiety/depression. EQ-5D includes five levels: 1 = “no difficulties,” 2 = “slight difficulties,” 3 = “moderate difficulties,” 4 = “serious difficulties,” and 5 = “extremely serious difficulties.”

### Psychometric assessments and statistical analysis

Kolmogorov–Smirnov test is used to test the normality of the total scores of BPI, WOMAC, and EQ-5D. Continuous variables were presented with mean values (SD), and categorical variables were presented with numbers (percentages). Statistical analyses were performed using SPSS 24.0 (IBM Corp., NY). The significance level was set to 0.05.

### Feasibility

Record any difficulties encountered by each participant in answering. Record the time required to complete the questionnaire.

### Floor and ceiling effects

The floor and ceiling effect is calculated by calculating the proportion of participants with the lowest or highest score of the BPI. If more than 15% of the participants reach the highest or lowest score, this indicates that the corresponding dimension has ceiling or floor effect [16].

### Reliability

#### Internal consistency and test–retest reliability

Reliability contains internal consistency and test–retest reliability. Internal consistency refers to the degree of correlation between items. It is measured by Cronbach's alpha, which should be greater than 0.7 under ideal conditions [22]. The test–retest reliability reflects the changes of the instrument in measuring the same patient without treatment. The test–retest reliability was evaluated by 50 patients who were randomly selected from the 150 participants. We sent the questionnaire again after an interval of 7 days. Test–retest reliability is detected by calculating the intraclass correlation coefficient (ICC), and ICC > 0.7 indicates good reliability.

#### Measurement error

Standard error of measurement (SEM) is an indicator of absolute reliability, reflecting the systematic and random error of the instrument [23]. SEM was measured using the formula $\mathrm{SEM}=\mathrm{SD}\times \surd \left(1-r\right)$, where SD is the pooled SD of BPI scores, and r is the test–retest reliability.

#### Construct validity

The simplified Chinese version of WOMAC and EQ-5D has been widely used in mainland China, and its validity and reliability were rigorously tested [24, 25]. The construct validity of BPI was assessed by the calculated Pearson coefficient of BPI-CV with WOMAC and EQ-5D. Correlations were judged as none or very weak (0–0.2), weak (0.21–0.4), moderate (0.41–0.60), strong (0.61–0.80), or very strong correlation (0.81–1.0) [4]. Before this analysis, we hypothesized that (1) EQ-5D and the pain dimension of WOMAC have a similar structure to the pain severity dimension of BPI, and (2) EQ-5D and the interference dimension of WOMAC have a good correlation with the functional interference dimension of BPI.

#### Structural validity

Because the factor structure of the BPI varies in different diseases, structure validity was investigated with an exploratory approach using an exploratory factor analysis (EFA) to assess the number of factors. The adequacy was calculated by the Kaiser–Meyer–Olkin test and Bartlett's sphericity test. Principal component analysis was normalized using the Kaiser maximum variance rotation method. The cutoff for load was set to 0.6.

## Results

### Participants

Detailed demographic and clinical characteristics of participants are shown in Table 1.

**Table 1:** Characteristics of participants

Characteristics	Total (*N* = 150)
Age (years, mean ± SD)	64.8 ± 6.1
*Sex, number* (%)	
Female	96 (64.0)
Male	54 (36.0)
Height (cm, mean ± SD)	161.9 ± 6.7
Weight (kg, mean ± SD)	67.8 ± 8.6
Body mass index (mean ± SD)	26.0 ± 2.8
*Educational level, number *(%)	
Primary school and below	69 (46.0)
Junior high school	45(30.0)
High school	29 (19.3)
University or above (including junior college)	7 (4.7)
*Residence status, number* (%)	
Live alone	14 (9.3)
Living with partner	98 (65.3)
Living with children	38 (25.3)
*Employment, number *(%)	
Farming	93 (62.0)
Unemployed	12 (8.0)
Retirement (including no longer farming)	45 (30.0)
Sickness time (years, mean ± SD)	4.7 ± 3.4
*Side, number* (%)	
Right	76 (50.7)
Left	74 (49.3)

### Feasibility

There were no missing data in any individual item of the BPI-CV or any of the other two scales. The time required to complete the BPI-CV was 4.9 min (SD 0.5).

### Floor and ceiling effects

Floor and ceiling effects for BPI-CV are shown in Table 2. Neither the lowest nor the highest score of BPI-CV was found in the present study. Therefore, no floor or ceiling effects were detected.

**Table 2:** The score distribution, ceiling and floor effects, and internal consistency reliability of BPI-CV

	Mean ± SD	% Floor^*1*^	%Ceiling^*2*^	Cronbach’s α^*3*^
BPI-CV	4.2 ± 1.4	0	0	0.894
Pain severity	3.7 ± 1.6	0	0	0.928
Function interference	4.6 ± 1.5	0	0	0.823

^*1*^% scoring worst possible value (0)

^*2*^% scoring best possible value (10)

^*3*^Internal consistency reliability

### Reliability

The Cronbach α of BPI-CV is shown in Table 2. For the BPI-CV, the total Cronbach α coefficients for all items were 0.894, which showed the homogeneity of the items included in the BPI-CV. Test–retest reliability was excellent, ICC of BPI-CV was 0.852 (95%CI, 0.785–0.905). SEM was 0.539 points.

### Construct validity

The correlations of BPI-CV with WOMAC and EQ-5D are shown in Table 3. The total BPI-CV score exhibits a very strong correlation of 0.827 with the WOMAC, indicating a significant association between overall pain assessment using the BPI-CV and the WOMAC questionnaire. The pain severity dimension had strong correlations with the WOMAC (*r* = 0.769), while moderate correlations were found with the EQ-5D (*r* = 0.527). The function interference dimension had strong correlations with both the WOMAC (*r* = 0.684) and the EQ-5D (*r* = 0.743). The emotional interference dimension had moderate correlations with the WOMAC (*r* = 0.512), while strong correlations were found with the EQ-5D (*r* = 0.650). The EQ-5D reveals a strong correlation of 0.768 with the BPI-CV, indicating a significant relationship between health-related quality of life and pain assessments.

**Table 3:** Pearson’s correlation coefficient between the Brief Pain Inventory (BPI-CV) with subscales of the WOMAC and the EQ-5D questionnaires for patients with osteoarthritis (*N* = 150)

	Pain severity	Activity interference	Emotional interference	Total BPI-CV
WOMAC	0.769^**^	0.684^**^	0.512^**^	0.827^**^
Pain	0.860^**^	0.689^**^	0.496^**^	0.862^**^
Stiffness	0.367^**^	0.223^**^	0.152^*^	0.311^**^
Physical function	0.712^**^	0.678^**^	0.517^**^	0.799^**^
EQ-5D	0.527^**^	0.743^**^	0.650^**^	0.768^**^

^**^Correlation is significant at the 0.01 level

^*^Correlation is significant at the 0.05 level

### Structural validity

The Kaiser–Meyer–Olkin (KMO) measure of sampling adequacy value was 0.852 with a statistically significant Barlett sphericity (*P* < 0.001). After exploratory factor analysis, it was loaded on three factors, namely, pain intensity, activity interference, and emotional interference. Table 4 shows the three factors and factor loadings for the BPI-CV items. The pain intensity factor included all four pain intensity items and the sleep item and accounted for 30.4% of the variance. The activity interference factors included three items: general activity, walking ability, and normal work, which accounted for 28.9% of the variance. Emotional interference included emotions, relationships with others, and enjoyment of life, accounting for 19.7% of the variance. These three factors cumulatively accounted for 79.0% of the total variance. Factor loadings for these three factors ranged from 0.638 to 0.913.

**Table 4:** Factor loadings of the factor analysis of the BPI-CV items rotated factor matrix

	Factor I	Factor II	Factor III
Pain worst	**0.638**^*1*^	0.598	0.016
Pain least	**0.860**	0.315	0.147
Pain average	**0.774**	0.546	0.070
Pain now	**0.896**	0.270	0.101
General activity	0.278	**0.775**	0.335
Mood	0.381	0.396	**0.642**
Walking ability	0.201	**0.845**	0.219
Normal work	0.090	**0.842**	0.304
Relationship	− 0.005	0.126	**0.913**
Sleep	**0.678**	− 0.110	0.216
Enjoyment of life	0.251	0.389	**0.767**

^*1*^Bold items represent loading on a factor

## Discussion

The present study translated BPI into a Chinese Version and culturally adapted, and the results showed that BPI-CV had good psychometric properties in patients with KOA.

Epidemiological studies have shown that symptomatic KOA affects 24% of the general population, and the main harms of KOA are pain and functional limitations, which are important factors for patients to decide on surgery when in pain [26]. The accurate evaluation of pain, which can provide a reference basis for the management of KOA patients and the development of targeted interventions, is of great clinical value [27].

BPI has been validated and adapted for several languages, including Chinese [28]. But so far, Chinese version of BPI has only been validated in patients with cancer pain. Although pain is a universal experience, patients with cancer pain versus non-cancer pain may differ in how they perceive pain and how the pain interferes with their lives. Some scholars compared BPI pain ratings of patients with typical nonmalignant pain and cancer pain and found that the former were more likely to rate their pain intensity at the top of the scale, whereas pain ratings of patients with cancer tended to be more evenly distributed across the various levels of the scoring scale [29]. Therefore, it cannot be assumed that the reliability and validity data of BPI scales in patients with cancer pain also support validity in other chronic pain patient populations. So, it is urgent to verify the reliability and validity of BPI in a large number of patients with nonmalignant pain, such as those with KOA.

There were no ceiling or floor effects in BPI-CV in the KOA patients, which is consistent with the results of the Norwegian version [4] and Spanish version of BPI [22], and indicates that the BPI-CV is a questionnaire with good discriminatory ability.

Internal consistency for all versions of the BPI was expressed using the Cronbach α coefficient. The Cronbach α coefficient for the BPI-CV in the present study was 0.894, which is generally consistent with the English version (0.86–0.96) [14], the Norwegian version (0.87–0.88) [4], the Spanish version (0.834–0.850) [22], the Turkish version (0.84–0.89) [5, 30], and the Persian version of BPI (0.88–0.91) [31]. All alpha values above indicated good internal consistency. The ICC of the present study was 0.852, which was basically consistent with the Turkish version (*r* = 0.77–0.88) [5, 30] and the Persian version of BPI (*r* = 0.87–0.91) [31].

In terms of construct validity, the results of the present study showed that the BPI-CV was moderately to highly correlated with the pain dimension (*r* = 0.496–0.860) and the functional interference dimension (*r* = 0.517–0.712) of the WOMAC and weakly correlated with the WOMAC stiffness dimension (*r* = 0.152–0.367), probably because the BPI mainly assesses pain and functional interference and does not assess stiffness with specific items. This observation result is basically consistent with the trend of the results observed in the Norwegian version [4], which confirms the rationality of the dimension classification used in the present study. There was a strong correlation between the total BPI-CV and EQ-5D (*r* = 0.768). All these results indicate that BPI-CV has qualified construct validity.

The structural validity of the BPI-CV was verified using exploratory factor analysis. The results of the Turkish version [5, 30] and Persian version [31] were consistent with the original version and contained two factors, four pain severity items and seven pain interference items. However, the interference items “sleep” and “enjoyment of life” in the English version did not load steadily on either factor [14]. Compared to the results of the English version, all factors in the present study were loaded stably with factor loadings greater than 0.6. However, the results of the present study revealed three factors, rather than two. There were studies that also reported a three-factor model in which the “sleep” item loaded on the emotional dimension, and in the present study, the “sleep” loaded on the pain dimension [32, 33]. It has been shown that osteoarthritis is associated with short sleep duration due to disease-related pain leading to disruption of sleep patterns [34]. This may be the reason why the “sleep” item loaded into the pain dimension.

Since BPI was originally developed to assess the pain severity and impact of cancer patients and evaluate the analgesic effectiveness for these patients, we have adapted the original BPI to better evaluate the pain status of KOA patients. First, the first item of the original BPI is to ensure that the scale is only used for cancer pain patients, while the present study is mainly used for KOA patients, so we decided to delete this item [35]. Secondly, the second item of the original BPI is to identify the specific site of cancer pain, while the present study focuses on KOA patients. Therefore, we use the partial picture of the knee joint instead of the whole-body picture. Thirdly, when using BPI, the setting of reference period is directly related to the purpose of the investigation, which may limit the collection of information and is the premise of pain assessment [35]. The reference period of the original version of BPI is only 24 h, but the pain of KOA patients is a chronic pain [36], and the reference period should be extended accordingly, so that the patient's pain status can be assessed more comprehensively [35]. Some studies have increased the reference period to 1 week and achieved satisfactory results [30]. Therefore, we decided to use “1 week” as the reference period of BPI.

Moreover, it is important to acknowledge that our research has certain limitations. Firstly, the lack of longitudinal data poses a challenge in accurately measuring responsiveness, such as determining the smallest clinically significant difference or impact size. Secondly, the patient sample primarily consists of individuals from central and western regions of China, which may not be fully representative of the entire Chinese population. To address these limitations, we aim to conduct a multi-center study in the future investigations.

In conclusion, we have successfully translated and adapted the BPI-CV instrument. The translated and adapted version has demonstrated good feasibility, reliability, and validity. The BPI-CV serves as a simple, valid, and reliable tool for assessing subjective experiences of pain severity, activity interference, and emotional interference in patients with KOA.

### Supplementary Information

**Additional file 1**. Chinese version of the Brief Pain Inventory.

## Data Availability

The datasets generated and/or analyzed during the current study are not publicly available because they are still being used in subsequent studies, but can be obtained from the corresponding author on reasonable request.

## Abbreviations

KOA: Knee osteoarthritis
PROMs: Patient-reported outcome measures scale
BPI: Brief Pain Inventory
BPI-CV: Chinese version of the Brief Pain Inventory
BMI: Body mass index
WOMAC: Western Ontario and McMaster Universities Osteoarthritis Index
EQ-5D: The EuroQoL Group's five-dimension questionnaire
HRQoL: Health-related quality of life
ICC: Intraclass correlation coefficient
SEM: Standard error of measurement
EFA: Exploratory factor analysis
KMO: Kaiser–Meyer–Olkin
