# Supplementary material for: Validation of the Chinese version of the Brief Pain Inventory in patients with knee osteoarthritis
Source: J Orthop Surg Res. 2023 Sep 23;18:720. doi: 10.1186/s13018-023-04218-1 (PMC10518095; doi:10.1186/s13018-023-04218-1)
Supplement: Supplementary file 1 — Additional file 1. Chinese version of the Brief Pain Inventory. [file 13018_2023_4218_MOESM1_ESM.docx]

**Chinese version of the Brief Pain Inventory:**

简明疼痛量表

1.请您在下图中标出您的疼痛部位，并在疼痛最剧烈的部位以“Ｘ”标出


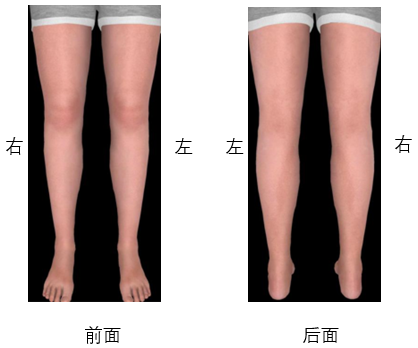


2. 请选择下面的一个数字，以表示过去一周内您**疼痛最剧烈**的程度。

（不痛）０ １ ２ ３ ４ ５ ６ ７ ８ ９ １０（最剧烈）

3. 请选择下面的一个数字，以表示过去一周内您**疼痛最轻微**的程度。

（不痛）０ １ ２ ３ ４ ５ ６ ７ ８ ９ １０（最剧烈）

4. 请选择下面的一个数字，以表示过去一周内您**疼痛的平均**程度。

（不痛）０ １ ２ ３ ４ ５ ６ ７ ８ ９ １０（最剧烈）

5. 请选择下面的一个数字，以表示您**目前的疼痛**程度。

（不痛）０ １ ２ ３ ４ ５ ６ ７ ８ ９ １０（最剧烈）

6. 您正在接受何种药物或治疗控制您的疼痛？

7. 在过去的一周内，由于药物或治疗的作用，您的疼痛缓解了多少？请选择下面的一个百分数，以表示疼痛缓解的程度。

（无缓解）0 10％ 20％ 30％ 40％ 50％ 60％ 70％ 80％ 90％ 100％（完全缓解）

8. 请选择下面的一个数字，以表示过去一周内疼痛对您的影响

（1）对日常生活的影响

（无影响）０ １ ２ ３ ４ ５ ６ ７ ８ ９ 10（完全影响）

（2）对情绪的影响

（无影响）０ １ ２ ３ ４ ５ ６ ７ ８ ９ 10（完全影响）

（3）对行走能力的影响

（无影响）０ １ ２ ３ ４ ５ ６ ７ ８ ９ 10（完全影响）

（4）对日常工作的影响（包括外出工作和家务劳动）

（无影响）０ １ ２ ３ ４ ５ ６ ７ ８ ９ 10（完全影响）

（5）对与他人关系的影响

（无影响）０ １ ２ ３ ４ ５ ６ ７ ８ ９ 10（完全影响）

（6）对睡眠的影响

无影响）０ １ ２ ３ ４ ５ ６ ７ ８ ９ 10（完全影响）

（7）对生活兴趣的影响

（无影响）０ １ ２ ３ ４ ５ ６ ７ ８ ９ 10（完全影响）
